# Geographic distribution of human leishmaniasis and phlebotomine sand flies in the State of Mato Grosso do Sul, Brazil

**DOI:** 10.1186/s13071-022-05353-0

**Published:** 2022-06-24

**Authors:** Herintha Coeto Neitzke-Abreu, Gabriel Barbosa Costa, Milena Nunes da Silva, Edith Palacio, Alexandre da Silva Cardoso, Paulo Silva de Almeida, Manoel Sebastião da Costa Lima-Junior

**Affiliations:** 1 Programa de Pós-Graduação em Ciências da Saúde, Faculdade de Ciências da Saúde, Universidade Federal da Grande Dourados, Dourados, MS Brazil; 2 Faculdade de Ciências Biológicas e Ambientais, Universidade Federal da Grande Dourados, Dourados, MS Brazil; 3 Laboratório Regional de Entomologia, Núcleo Regional de Saúde, Dourados, MS Brazil; 4 Laboratório de Imunopatologia e Biologia Molecular, Instituto Aggeu Magalhães/Fiocruz, Recife, PE Brazil

**Keywords:** Visceral leishmaniasis, Cutaneous leishmaniasis, Sand flies, Disease notification, Information system, Vectors

## Abstract

**Background:**

In the State of Mato Grosso do Sul, Brazil, sand flies and cases of visceral (VL) and cutaneous (CL) leishmaniases have been reported in almost all municipalities. The aim of this study was to analyze the geographic distribution of VL and CL in relation the sand fly species found in the municipalities of Mato Grosso do Sul.

**Methods:**

We analyzed VL and CL cases from 2001 to 2018 using data from the Notifiable Diseases Information System (SINAN). Data collected since 2003 on the presence of sand fly vectors (proven or suspected) were provided by the State Health Secretariat.

**Results:**

A total of 3566 and 3030 cases of VL and CL, respectively, were reported from 2001 to 2018. The municipalities with the most reported cases of VL were Campo Grande (2495), Três Lagoas (442), Corumbá (140) and Aquidauana (136); and those for CL were Campo Grande (635) and Bodoquena (197). The following sand fly species with vector potential were found in 59 municipalities (74.7%): *Lutzomyia longipalpis*, *Lutzomyia cruzi*, *Nyssomyia whitmani*, *Migonemyia migonei*, *Nyssomyia neivai*, *Pintomyia pessoai*, *Bichromomyia flaviscutellata* and *Pintomyia fischeri*. Sand flies were present in six municipalities where no cases of VL were reported and in two municipalities where no cases of CL were reported.

**Conclusions:**

Our results indicate that the geographical distribution of VL and CL in Mato Grosso do Sul expanded during the study period, and highlight the presence of sand fly vectors in municipalities where these diseases are currently considered to be non-endemic.

**Graphical Abstract:**

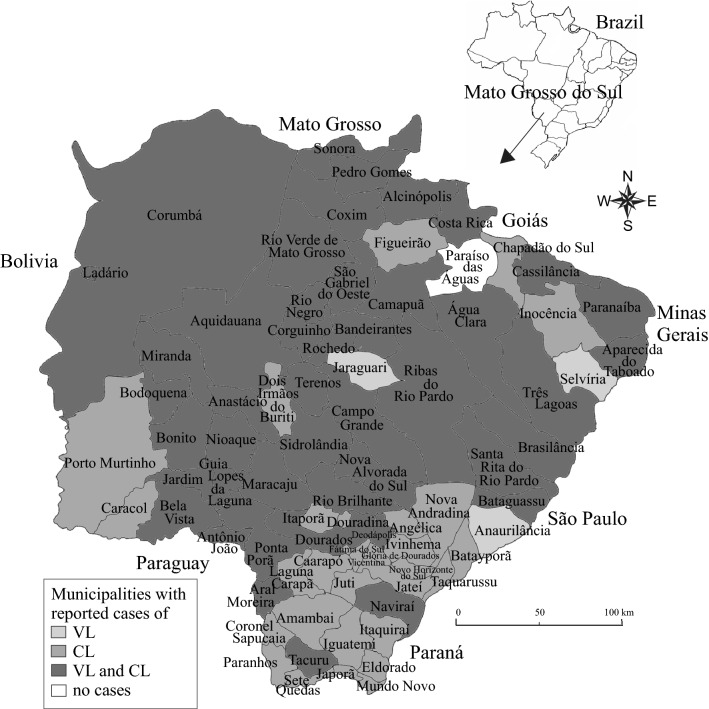

## Background

Leishmaniases are a group of diseases caused by protozoa of the genus *Leishmania*, which are transmitted by the bite of female phlebotomine sand flies. There four main clinical forms of leishmaniasis: visceral leishmaniasis (VL), post-kala-azar dermal leishmaniasis (PKDL), cutaneous leishmaniasis (CL) and mucocutaneous leishmaniasis (MCL) [1]. Among these, VL and CL are the most common clinical forms. VL is the most severe clinical form of the disease, being potentially fatal even in patients receiving treatment. CL ranges clinically from localized skin ulcers to diffuse painless dermal nodules, sometimes evolving to MCL, resulting in ulceration of the oropharynx [2]. Several animals (wildlife and synanthropes) can act as reservoirs of various *Leishmania* spp., but domestic dogs play a major role as the main reservoir of *Leishmania infantum*, the causative agent of zoonotic VL [3].

Leishmaniases are endemic in Brazil, which is the largest focus of both VL and CL in the Americas [4]. Among the states in the midwest region of Brazil, Mato Grosso do Sul has the highest VL incidence, whereas Mato Grosso has the highest number of CL cases [5]. Moreover, the lethality of VL in Mato Grosso do Sul is higher than the national average [6]. These data emphasize the need for studies on the epidemiology of both VL and CL in this Brazilian state, which may provide information on the expansion of these diseases in Brazil. In this context, the objective of this study was to describe the geographical distribution of VL and CL cases in the State of Mato Grosso do Sul from 2001 and 2018. The presence of sand fly vectors in endemic and non-endemic municipalities was also evaluated.

## Methods

### Study area

Taken together, the municipalities of the State of Mato Grosso do Sul, located in the Midwest Region of Brazil, have a total territorial area of 357,145.534 km^2^. Mato Grosso do Sul is bordered to the east by the States of Minas Gerais and São Paulo, to the south by Paraná State and to the north by the States of Mato Grosso and Goiás, and it has international borders with Bolivia and Paraguay to the west. The state comprises 79 municipalities, divided into 11 microregions, with almost 3 million inhabitants [7].

Depending on the region, Mato Grosso do Sul has the following Köppen climatic classification: Am (coldest temperature and short dry season), Aw (rainy season in summer and dry season in winter), Cfa (hot summers, infrequent frosts, and more concentrated rains in the summer) and Af (no defined dry season) [8].

### Data collection

A descriptive study was carried out using secondary data collected on all recorded VL and CL cases in all municipalities of Mato Grosso do Sul between 2001 and 2018. The municipality in which the VL and CL cases were notified (notification municipality) and the infection site were analyzed, using information registered in the Notifiable Diseases Information System (SINAN) and made available in Datasus. The notification municipality is the city where the patient was diagnosed with leishmaniasis and subsequently treated. The infection site is the municipality where the patient resides and where the infection probably occurred.

Data on the collection of sand flies (proven/suspected vector species) were provided by the Entomology Coordination of Dourados of the State Health Secretariat in January 2021. The collection of sand flies was carried out using CDC light traps, between 2003 and 2020. In brief, collections were performed during 3 consecutive nights in approximately 10 sites per municipality (according to local operational capacity). Collection sites were in peridomicile structures (e.g. chicken coops, pigsties and stables) and presented suitable conditions for sand flies. On some occasions (i.e. when VL cases were reported), entomological investigation was also carried out intradomicile. Sand fly identification was performed using the morphological keys proposed by Shimabukuro [9]. In this work, we followed Galati’s nomenclature [10] and used abbreviations of genera and subgenera as proposed by Marcondes [11].

## Results

**Fig. 1:**
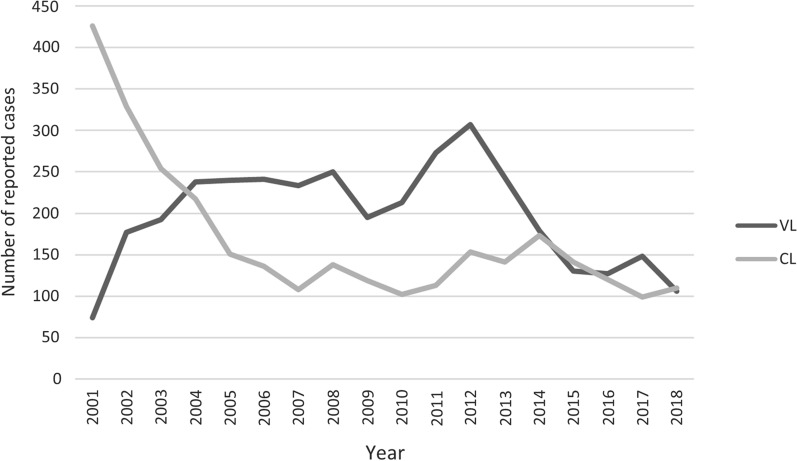
Number of visceral and cutaneous leishmaniasis cases, 2001–2018, reported in the State of Mato Grosso do Sul, Brazil. Data from the Notifiable Diseases Information System (SINAN). Abbreviations: CL, Cutaneous leishmaniasis; VL, visceral leishmaniasis

A total of 6596 cases of leishmaniasis were reported from 2001 to 2018 in Mato Grosso do Sul State, of which 3566 were VL and 3030 were CL (Table 1). The average incidence was 35.7 cases/100,000 inhabitants. The years with the most VL cases were 2012 (*n* = 307) and 2011 (*n* = 273), with a decrease in the number of cases between 2014 and 2018. Most of the CL cases were reported in 2001 (*n* = 426) and 2002 (*n* = 329), with a decrease in the number of reported cases in 2010 and 2017 (Fig. 1).

**Table 1:** Number of VL and CL cases in municipalities identified as notification site or infection site, and presence of sand fly vectors, during 2001–2018, Mato Grosso do Sul State, Brazil

Municipality	Notification site^a^		Infection site^b^		Species of sand flies^c^							
	VL	CL	VL	CL	1	2	3	4	5	6	7	8
Água Clara	8	5	15	9	x	–	x	–	x	–	–	–
Alcinópolis	2	36	3	35	x	x	x	–	–	–	–	–
Amambai	0	38	2	19	–	–	–	–	–	–	–	–
Anaurilândia	1	0	0	1	–	–	–	–	–	–	–	–
Angélica	0	22	1	9	–	–	–	–	–	–	–	–
Anastácio	72	25	89	16	x	–	x	–	–	–	–	–
Antônio João	4	1	9	2	x	–	x	x	–	x	–	–
Aparecida do Taboado	1	4	3	2	x	–	x	–	–	–	–	–
Aquidauana	136	123	145	102	x	–	x	–	x	x	x	–
Aral Moreira	2	13	2	10	–	–	–	–	–	–	–	–
Bandeirantes	1	3	3	4	x	–	–	–	–	–	–	–
Bataguassu	8	6	9	6	x	–	x	x	x	x	–	x
Batayporã	0	8	0	4	–	–	–	–	–	–	–	–
Bela Vista	6	13	15	11	x	–	x	–	–	–	x	–
Bodoquena	2	197	11	161	x	–	x	x	x	–	–	–
Bonito	10	94	29	49	x	x	x	–	x	–	x	–
Brasilândia	5	1	28	2	x	–	x	–	x	–	–	–
Caarapó	0	21	0	9	–	–	x	x	–	x	–	x
Camapuã	5	14	14	23	–	x	x	–	–	–	–	–
Campo Grande	2495	635	1843	197	x	x	x	x	–	–	x	–
Caracol	0	6	1	6	x	–	x	–	–	–	–	–
Cassilândia	3	40	3	12	–	x	–	–	–	–	–	–
Chapadão do Sul	0	9	3	4	–	–	–	–	–	–	–	–
Corguinho	2	5	2	22	x	–	x	–	–	–	–	–
Coronel Sapucaia	0	19	0	8	–	–	–	–	–	–	–	–
Corumbá	140	32	152	27	–	x	–	–	–	–	–	–
Costa Rica	2	55	3	32	x	–	x	–	–	–	–	–
Coxim	31	100	63	73	x	–	x	–	–	–	–	–
Deodápolis	2	22	0	16	–	–	–	–	–	–	–	–
Dois Irmãos do Buriti	0	24	15	10	x	–	x	–	–	–	–	–
Douradina	0	5	0	1	–	–	–	–	–	–	–	–
Dourados	36	74	19	18	x	–	x	x	x	x	x	–
Eldorado	0	23	1	13	–	–	–	–	–	–	–	–
Fátima do Sul	0	4	0	4	–	–	–	–	–	–	–	–
Figueirão	0	5	2	6	–	–	–	–	–	–	–	–
Glória de Dourados	0	2	0	3	–	–	–	–	–	–	–	–
Guia Lopes da Laguna	4	65	13	41	x	–	x	–	x	x	–	–
Iguatemi	0	37	0	22	–	–	–	–	–	–	–	–
Inocência	0	5	2	3	x	–	x	–	–	–	–	–
Itaporã	0	15	1	7	–	–	x	–	–	–	–	–
Itaquiraí	0	50	1	46	–	–	–	–	x	–	–	–
Ivinhema	0	27	0	25	–	–	–	–	–	–	–	–
Japorã	0	12	0	9	–	–	x	x	x	–	–	–
Jaraguari	1	0	3	5	x	–	x	–	–	–	–	–
Jardim	24	39	55	27	x	–	x	x	–	–	–	–
Jateí	0	3	0	3	–	–	–	–	–	–	–	–
Juti	0	3	0	5	–	–	–	–	–	–	–	–
Ladário	6	7	31	5	–	x	x	–	–	–	–	–
Laguna Carapã	0	4	0	11	–	–	x	–	–	–	–	–
Maracajú	5	125	14	54	x	–	x	–	–	x	–	–
Miranda	14	42	40	31	x	–	x	–	–	–	–	–
Mundo Novo	0	20	0	13	–	–	x	–	x	–	–	–
Naviraí	1	99	2	66	–	–	x	–	x	–	–	–
Nioaque	2	79	12	76	x	–	x	–	x	–	–	–
Nova Alvorada do Sul	1	4	5	9	x	–	x	–	–	–	–	–
Nova Andradina	0	48	1	23	–	–	x	–	x	x	–	x
Novo Horizonte do Sul	0	51	0	42	–	–	x	–	x	x	–	–
Paranaíba	3	28	3	14	x	–	x	–	–	–	–	–
Paranhos	0	36	0	11	x	–	x	–	–	–	–	–
Pedro Gomes	2	139	5	126	x	–	x	–	–	–	x	–
Ponta Porã	15	65	24	30	x	–	x	–	–	x	–	–
Paraíso das Águas	0	0	0	0	x	x	–	–	–	–	–	–
Porto Murtinho	0	10	6	32	x	–	x	–	–	–	–	–
Ribas do Rio Pardo	10	11	32	15	x	–	x	–	–	–	–	–
Rio Brilhante	2	13	2	12	–	–	x	x	–	x	–	–
Rio Negro	4	10	14	17	x	–	x	–	–	–	–	–
Rio Verde de Mato Grosso	35	81	84	75	x	–	x	x	x	–	x	–
Rochedo	3	3	8	9	x	–	x	–	x	–	–	–
Santa Rita do Pardo	2	3	0	5	x	–	x	–	x	x	x	–
São Gabriel do Oeste	2	2	7	12	x	x	x	–	–	–	x	–
Selvíria	2	0	6	0	x	–	x	–	–	–	–	–
Sete Quedas	0	36	1	17	–	–	–	–	–	–	–	–
Sidrolândia	2	10	13	17	x	–	x	–	–	–	–	–
Sonora	1	74	1	61	x	–	x	–	–	–	x	–
Tacuru	1	40	1	38	–	–	x	–	x	–	–	–
Taquarussu	0	1	1	3	–	–	–	–	–	–	–	–
Terenos	8	15	40	22	x	–	x	–	x	–	–	–
Três Lagoas	442	33	470	22	x	–	x	–	x	–	x	–
Vicentina	0	1	0	0	–	–	–	–	–	–	–	–
TOTAL	3566	3030	3383	1987	44	9	54	20	12	11	10	3

*CL* cutaneous leishmaniasis,* VL* visceral leishmaniasis

^a^Notification site is the where the patient was diagnosed with leishmaniasis and subsequently treated

^b^Infection site is the municipality where the patient resides and where the infection probably occurred

^c^1, *Lutzomyia longipalpis*; 2, *Lutzomyia cruzi*; 3, *Nyssomyia whitmani*; 4, *Migonemyia migonei*; 5, *Nyssomyia neivai*; 6, *Pintomyia pessoai*; 7, *Bichromomyia flaviscutellata*; 8, *Pintomyia fischeri*; x, vector presence

The municipalities in which the highest number of total cases (VL and CL combined) were reported were Campo Grande (*n* = 3,130), Três Lagoas (*n* = 475), Aquidauana (*n* = 259), Bodoquena (*n* = 199), Corumbá (*n* = 172), Pedro Gomes (*n* = 141), Coxim (*n* = 131) and Maracaju (*n* = 130). The highest number of VL cases were reported in the municipalities of Campo Grande (*n* = 2495), Três Lagoas (*n* = 442), Corumbá (*n* = 140), Aquidauana (*n* = 136), Anastácio (*n* = 72) and Dourados (*n* = 36). The highest number of CL cases were reported in the municipalities of Campo Grande (*n* = 635), Bodoquena (*n* = 197), Pedro Gomes (*n* = 139), Maracajú (*n* = 125), Aquidauana (*n* = 123) and Coxim (*n* = 100) (Table 1).

It was possible to trace the original infection site in 3383 cases of VL and 1987 cases of CL. The municipalities most frequently cited as a infection site of VL cases were Campo Grande (*n* = 1843), Três Lagoas (*n* = 470), Corumbá (*n* = 152), Aquidauana (*n* = 145) and Anastácio (*n* = 89). The most frequently reported infection sites for CL were Campo Grande (*n* = 197), Bodoquena (*n *= 161), Pedro Gomes (*n *= 126), Aquidauana (*n *= 102) and Rio Verde do Mato Grosso (*n *= 75) (Table 1).

**Fig. 2:**
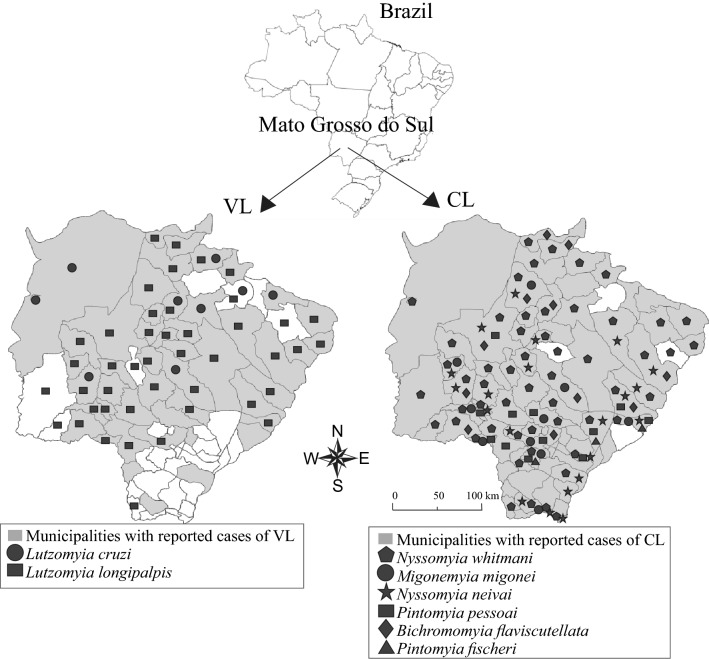
Municipalities with reported cases of VL and CL and where sand flies were reported to be present, 2001–2018, Mato Grosso do Sul, Brazil. Data on cases are from the SINAN and sand fly data (collected since 2003) are from the State Health Secretariat

Of the 79 municipalities of Mato Grosso do Sul, 59 (74.7%) reported the presence of at least one of the following proven/suspected sand fly vectors: *Bichromomyia flaviscutellata*, *Lutzomyia longipalpis*, *Lutzomyia cruzi*, *Migonemyia migonei*, *Nyssomyia neivai*, *Nyssomyia whitmani*, *Pintomyia fischeri* and *Pintomyia pessoai* (Fig. 2).

*Lutzomyia longipalpis* was the most widely distributed vector species, being reported in 44 municipalities, whereas *Lu. cruzi* was reported in nine municipalities. *Nyssomyia whitmani* (54 municipalities) was the predominant CL vector, followed by *Ny. neivai* (21 municipalities), *Pi. pessoai* (12 municipalities), *Bi. flaviscutellata* (11 municipalities), *Mi. migonei* (10 municipalities) and *Pi. fischeri* (three municipalities) (Fig. 2).

Detailed information on the reported VL and CL cases, original infection sites as well as the presence of proven/suspected sand fly vectors in all municipalities is summarized in Table 1. A total of 15 municipalities had no VL cases or proven/suspected vectors and were not identified as infection sites. Eight municipalities had no reported VL cases or proven/suspected vectors, but were identified as infection sites. Two municipalities had no reported VL cases and were not identified as infection sites, but proven/suspected vectors were present (Table 1).

Regarding CL, only one municipality (Paraíso das Águas) had no CL cases or proven/suspected vectors and was not identified as infection site. Anaurilândia had no CL cases or proven/suspected vectors, but was identified as an infection site. Jaraguari had no CL cases, but was identified as infection site and had proven/suspected vectors present. Finally, Selvíria had no CL cases nor was it identified as infection site, but had proven/suspected vectors (Table 1).

## Discussion

Visceral leishmaniasis is traditionally endemic in Mato Grosso do Sul State, but until a number of decades ago the disease was restricted in terms of geographical distribution, with its distribution from 1913 to 1993 limited to the municipalities of Corumbá and Ladário. With the construction of the Bolivia-Brazil gas pipeline, the railroad and federal highway BR-262, VL extended its distribution in Mato Grosso do Sul and reached other cities/municipalities, such as Campo Grande, Três Lagoas and Aquidauana [12]. Data summarized in this study demonstrate a further expansion of VL, which now occurs in 62.0% (49/79) of the municipalities of Mato Grosso do Sul. CL is even more widespread in this state, with cases reported in 94.9% (75/79) of the municipalities; two other municipalities have also been identified as infection sites for reported CL cases.

The expansion of VL in Mato Grosso do Sul could be related to several factors, including human population movements, environmental changes, poor housing and nutritional conditions of the populations at risk, widespread presence of *Lu. longipalpis* and absence of systematic vector control measures [12, 13]. This study also highlights that many patients needed to go to another municipality to seek healthcare services, which may potentially result in delayed diagnosis and treatment [14]. Indeed, in many cases, the municipality responsible for the notification differed from the original infection site.

Only four municipalities were identified as not reporting CL cases: Anaurilândia, Jaraguari, Paraíso das Águas and Selvíria. This absence of reported CL cases may be due to the absence of the disease in these municipalities or to underreporting. All four of these municipalities are bordered by municipalities with reported cases, which certainly suggests that there is a risk for CL introduction, if it not already present. Indeed, Anaurilândia and Jaraguari were identified as the probable original infection sites.

The notification forms assessed in this study were incomplete for many reported VL/CL cases, with at least one piece of missing information. This problem has also been reported by other researchers [15], which highlights the need for improving both data capture form compilation and data entry in SINAN.

The occurrence of VL and CL cases in most municipalities of Mato Grosso do Sul State indicates the presence of sand fly vectors in these municipalities. Of 71 sand fly species registered in Mato Grosso do Sul [16], eight are considered to be of medical significance [16, 17].

Regarding the vectors of *Leishmania infantum* (VL), there was a predominance of *Lu. longipalpis*, followed by *Lu. cruzi*, corroborating the findings of other studies [13, 18]. Our results suggest the expansion of the distribution of *Lu. longipalpis* in Mato Grosso do Sul State. Barrios et al. [16] did not detect the presence of *Lu. longipalpis* in Bandeirantes, São Gabriel do Oeste and Sonora municipalities. This geographical expansion of this vector may be influenced by deforestation [20] and agricultural enterprises [21], both human activities which may lead to an expansion of VL in the state. *Migonemyia migonei* (a permissive vector of *L. infantum* [22]) was also found in municipalities with cases of VL. Regarding vectors of *Leishmania braziliensis* and *Leishmania amazonensis* (causative agents of CL), there was a predominance of *Ny. whitmani* and *Ny. neivai* (25.3%), as reported previously [13, 19, 23].

Further entomological studies are needed in municipalities with reported cases but where with no sand fly vectors have been reported so far. The use of different trapping methods, with a higher number of traps and the installation of traps in different locations, may revel the presence of sand fly vectors in these municipalities. In the same way, healthcare professionals working in municipalities with a confirmed presence of sand fly vectors but with no leishmaniasis cases should be advised to actively search out possible new cases. This is pivotal to identifiy possible failures in the notification system and also to provide early diagnosis and treatment for affected patients.

## Conclusion

Our study indicates that the geographical distribution of VL and CL in Mato Grosso do Sul State during the study period has expanded, and highlights the presence of sand fly vectors in municipalities where these diseases are currently considered to be non-endemic.

## Abbreviations

CL: cutaneous leishmaniasis
VL: visceral leishmaniasis
